# Impact of hypercoagulable state on Crohn’s disease severity and progression: transcriptomic and single-cell analyses of the ileum

**DOI:** 10.3389/fimmu.2025.1611114

**Published:** 2025-09-25

**Authors:** Fengfei Wu, Fangting Wu, Hui Yang, Wenting Xie, Sinan Zhang, Lihua Zhou, Fang Xie, Lan Bai, Miaoxing Huang, Side Liu

**Affiliations:** ^1^ Guangdong Provincial Key Laboratory of Gastroenterology, Institute of Gastroenterology of Guangdong Province, Department of Gastroenterology, Nanfang Hospital, Southern Medical University, Guangzhou, China; ^2^ Guangdong Provincial Key Laboratory of Single-cell and Extracellular Vesicles, Southern Medical University, Guangzhou, China; ^3^ School of Public Health, Guilin Medical University, Guilin, China; ^4^ Department of Gastroenterology, The Tenth Affiliated Hospital, Southern Medical University (Dongguan People's Hospital), Dongguan, China

**Keywords:** Crohn's disease, hypercoagulable state, stem cell, CCR6, PI3K–Akt signaling pathway

## Abstract

**Background:**

The mechanisms linking hypercoagulability to disease severity in Crohn’s disease (CD) remain poorly understood. Through integrated transcriptomic and single-cell analyses of ileal tissues, we identified a novel CCR6^+^OLFM4^+^ intestinal stem cell subpopulation that bridges coagulation and inflammation in CD.

**Methods:**

A cohort of 78 CD patients was established, utilizing transcriptomic data from three independent ileal samples obtained from the GEO database as discovery and validation datasets. Coagulation-related DEGs (CRGs) were determined via AmiGO 2 and KEGG databases. Based on these CRGs, CD patients were subclustered, coagulation scores were calculated, and gene expression changes were evaluated. Public single-cell RNA sequencing data from CD patient ileal epithelial cells were analyzed to identify key target cells influenced by coagulation. Immune infiltration was evaluated based on coagulation scores across subgroups. Ileal tissues from CD patients with different coagulation statuses were examined using Immunofluorescence Staining.

**Results:**

Single-cell analysis of ileal epithelium revealed a novel CCR6^+^OLFM4^+^ stem cell subpopulation that was significantly expanded in CD patients with hypercoagulability (*P*<0.05). These cells showed marked upregulation of PI3K-Akt signaling and correlated strongly with disease severity. Immunofluorescence validation confirmed a 2.3-fold increase in CCR6^+^OLFM4^+^ cells in the epithelial layer of hypercoagulable CD patients compared to normocoagulable controls. The concurrent activation of coagulation pathways and immune cell infiltration in CD ileum suggests this stem cell subpopulation may serve as a critical link between hypercoagulability and disease progression.

**Conclusion:**

Our findings nominate CCR6^+^OLFM4^+^ stem cells as cellular mediators of coagulation-associated CD progression, suggesting the CCR6-PI3K-Akt axis as a potential therapeutic target requiring validation in larger cohorts.

## 1 Introduction

The interplay between coagulation and innate immunity has become a focal point in the study of inflammatory diseases, particularly Crohn’s disease (CD), a chronic systemic inflammatory disorder of the gastrointestinal tract (1–3). Traditionally studied as two separate systems, coagulation and immune responses are now recognized as tightly integrated processes, where their balance is critical to the body’s defense against injury and infection (4). However, in CD, this balance is often disrupted, leading to disease progression characterized by unpredictable cycles of remissions and relapses, as well as severe complications (1, 5).

Emerging evidence has shown that patients with CD are in a state of acquired hypercoagulability, which predisposes them to thromboembolic events such as deep vein thrombosis and pulmonary embolism (6, 7). This hypercoagulable state is particularly pronounced in patients with more severe disease, those on immunosuppressive therapy, such as corticosteroids, and those experiencing extended periods of immobility (8, 9). Interestingly, the inflammatory milieu in CD exacerbates the thrombotic risk by promoting endothelial dysfunction and platelet activation, creating a vicious cycle of inflammation and coagulation (10).

Thromboembolic events in CD are not only a major cause of morbidity and mortality, but they also contribute to exacerbating disease severity (11). Recent studies suggest that microthrombosis within the intestinal microvasculature could further worsen local inflammation, potentially leading to fibrosis and increased disease complications (12). Understanding the molecular mechanisms linking coagulation and inflammation in CD is therefore critical to developing targeted therapies that can mitigate both thrombotic events and intestinal inflammation.

Given the complexity of this relationship, recent efforts have sought to explore therapeutic strategies that modulate coagulation to restore immune homeostasis. For instance, antiplatelet agents such as phosphodiesterase inhibitors (e.g., dipyridamole) have shown promise in reducing inflammation and improving disease outcomes both in preclinical models of CD and in a small clinical trial involving pediatric CD patients (13). However, the precise molecular mechanisms underlying this link remain unclear, and further research is needed to uncover the exact role of coagulation pathways in CD pathogenesis.

In this study, we aim to address this gap by leveraging publicly available transcriptomic and single-cell RNA sequencing data from ileal biopsies of CD patients. Specifically, we will: 1) identify gene expression changes in ileal tissue associated with coagulation pathways, and 2) investigate how these changes correlate with disease severity and progression. By elucidating the molecular networks that connect coagulation with inflammatory processes in CD, we hope to identify novel biomarkers for disease monitoring and potential therapeutic targets.

## 2 Methods

### 2.1 Study participants

**Figure 1:**
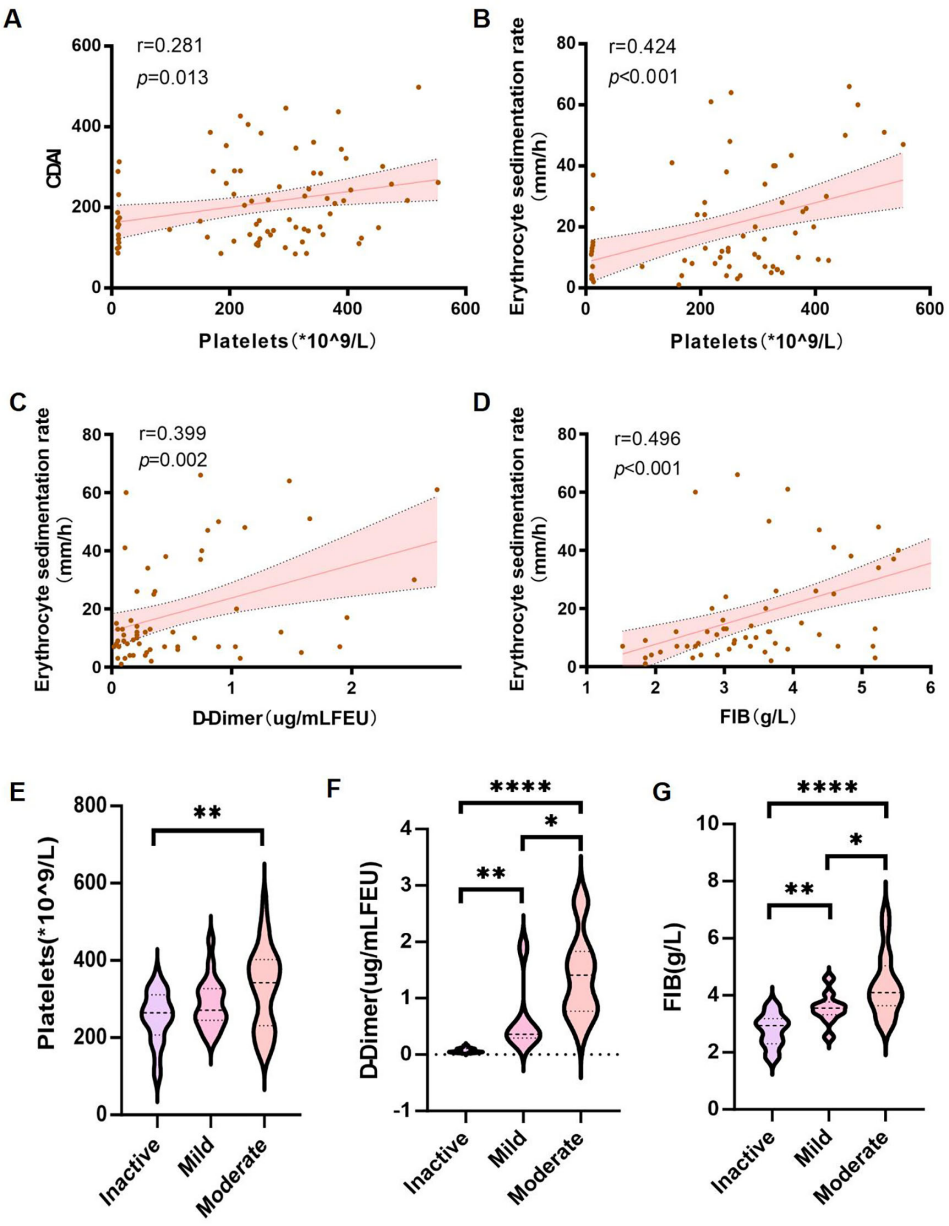
Association of coagulation markers with disease severity in CD patients. **(A–D)** Spearman’s correlation analysis between: **(A)** platelet count (PLT) and CDAI; **(B)** PLT and ESR; **(C)** D-dimer and ESR; **(D)** fibrinogen (FIB) and ESR. **(E–G)** Wilcoxon rank-sum tests comparing: **(E)** PLT; **(F)** D-dimer; and **(G)** FIB levels across inactive, mild, and moderate disease stages. Error bands represent 95% confidence intervals. *P< 0.05, **P < 0.01, and ****P < 0.001 indicate statistical significance.

78 patients with CD were recruited at Nanfang Hospital (Guangzhou, China). The diagnosis of CD was established using typical criteria (14). This study was performed following the ethical guidelines of the Declaration of Helsinki and was approved by the local ethical committee (NFEC-2024-199). Informed consent was obtained from all enrolled patients before collecting clinical information. This clinical dataset was used exclusively to establish initial associations between coagulation markers and disease severity (**Figure 1**).

### 2.2 Acquisition of public datasets and coagulation-related genes

Clinical samples were primarily utilized for validating coagulation-inflammation correlations due to limitations in RNA integrity for transcriptomic assays. For in-depth molecular analyses, three datasets (GSE193677, GSE137344 and GSE186582) were obtained from the Gene Expression Omnibus (GEO) (https://www.ncbi.nlm.nih.gov/geo/) database, {Kong, 2023 #1}which included gene expression profiles and corresponding clinical information for CD and controls. The discovery dataset was pooled with GSE193677 dataset and GSE137344 dataset, containing 234 CD ileal samples and 142 control samples. The GSE186582 dataset, containing 196 CD ileal samples, was utilized for validation. Probe IDs or ensembl gene IDs were transferred into gene symbols according to the corresponding annotation file, and all the data was transformed into log2 form for subsequent analyses.

We collected 147 genes related to coagulation from AmiGO 2 which is an official web-based set of tools for searching and browsing the Gene Ontology database (http://amigo.geneontology.org/amigo). We obtained 203 genes related to coagulation in the two coagulation pathways subsequently, including hsa04610 (Complement and coagulation cascades) and hsa04611 (Platelet activation), which were found in the KEGG database (https://www.genome.jp/kegg/). Finally, a total of 300 coagulation-related genes (CRGs) list was obtained by merging the two gene lists.

### 2.3 Identification of differentially expressed genes associated with CD and control

The “limma” R package was employed for performing the differential genes (DEGs) analysis between CD and control samples, applying adj. *P <*0.05 and |log2 Fold Change (FC)|>0.5 as threshold. Differential gene expression data was shown using volcano plots. Then the “clusterProfiler” R package were used for Gene Ontology (GO) enrichment analysis and Kyoto Encyclopedia of Genes and Genomes (KEGG) pathway analysis for further investigating the biological roles of DEGs.

### 2.4 Consensus clustering analysis of coagulation-related genes

We identified CRGs by merging 147 genes from AmiGO2 and 203 genes from KEGG pathways (hsa04610, hsa04611), yielding 300 unique CRGs. After intersecting these with DEGs from the discovery dataset, we obtained 64 overlapping CRGs that were both coagulation-related and differentially expressed in CD. These 64 CRGs were used for clustering analysis as they represent the most biologically relevant coagulation-associated genes dysregulated in CD. The unsupervised clustering “Pam” method based on Euclidean and Ward’ s linkage was applied subsequently to identify distinct molecular subtypes based on CRGs expression levels. Using the “ConsensusClusterPlus” R package, an unsupervised hierarchical clustering analysis was performed with 1000 iterations and an 80% resample rate, dividing both the discovery dataset and the validation dataset into two subclusters, respectively. Principal component analysis (PCA) was performed to show the distribution difference of coagulation subtypes.

### 2.5 Definition and calculation of coagulation score

To quantify coagulation pathway activity, we calculated a “Hallmark_coagulation score” using the “HALLMARK_COAGULATION” gene set from MSigDB (15). While our curated 300-CRG list was comprehensive, the HALLMARK set was selected for scoring because it is a standardized, experimentally validated collection of core coagulation genes optimized for GSVA. This approach minimizes batch effects and allows direct comparison with other studies using MSigDB benchmarks. “Hallmark_coagulation score” was calculated by “GSVA” R package, and a high score represents increased expression of pro-coagulation genes.

### 2.6 Subclusters analysis with CRGs

Principal component analysis (PCA) was performed to show the distribution difference of coagulation-related subclusters. Based on the clinical data of samples, mainly IBD_endoseverity of 4 levels (inactive, mild, moderate, severe) were expressed between the two clusters. The “limma” R package was employed for performing the DEGs analysis between Cluster 1 and Cluster 2, applying adj. *P <*0.05 and |log2 Fold Change (FC)|>0.5 as threshold. Differential gene expression data was shown using volcano plots. Then the “clusterProfiler” R package were used for GO enrichment analysis and KEGG pathway analysis for further investigating the biological roles of DEGs.

### 2.7 Gene set variation analysis

GSVA is a nonparametric and unsupervised method for evaluating enrichment of transcriptome gene set. By comprehensively scoring the gene set of interest, GSVA converts gene-level changes into pathway-level changes, and then judges the biological function of the sample. This study downloaded gene sets from the molecular signatures database, and the GSVA algorithm was used to comprehensively score each gene set, so as to evaluate the potential biological function changes of different samples.

### 2.8 Development of LASSO-derived diagnostic model

LASSO regression was used to generate the diagnostic mode. LASSO regression is known to be able to remove unimportant variables via the regression coefficients penalizing the size of the parameters. Applying the LASSO regression method, feature selection and predictive signature building was done. LASSO regression shrinks the coefficient estimates toward zero, with the degree of shrinkage dependent on an additional parameter, ℷ. To determine the optimal values for ℷ, a 10-time cross-validation was used, and we chose ℷ via the minimum criteria. The LASSO model is designed to determine whether a patient with CD has a hypercoagulable state and is the linear predictor of the binary model built on the training set with selected variables via LASSO algorithm.

### 2.9 Evaluating the immune cell infiltration

The gene expression datasets of the discovery dataset was prepared in accordance with the accepted format of CIBERSORT, and then data were uploaded to the CIBERSORT web portal (http://cibersort.stanford.edu/). We used the original CIBERSORT gene signature file LM22, which defines 22 immune cell subtypes, to analyze datasets from ileal tissues of CD patients and healthy controls. CIBERSORT *P*-value < 0.05 was included.

### 2.10 Single-cell RNA sequencing analysis

Publicly available single-cell RNA sequencing data of ileal epithelial cells from CD patients and healthy controls were obtained from the Broad DUOs repository (Accession DUOS-000146). After quality control and normalization, a total of 154,136 high-quality epithelial cells were retained for downstream analysis. Cell clustering was performed using the Seurat package (v4.0) based on principal component analysis (PCA) and uniform manifold approximation and projection (UMAP) for dimensionality reduction. Cell types were annotated according to established marker genes. Four distinct OLFM4^+^ stem cell subpopulations were identified: stem cells OLFM4, stem cells OLFM4 GSTA1, stem cells OLFM4 LGR5, and stem cells OLFM4 PCNA. Differential gene expression analysis between inflammatory and non-inflammatory samples was conducted using the MAST framework. Gene Set Variation Analysis (GSVA) was applied to evaluate the enrichment of coagulation and PI3K-Akt signaling pathways across stem cell subtypes. Pseudotime trajectory analysis was performed using Monocle3 to infer cellular differentiation dynamics. Immunofluorescence staining of ileal tissue sections was used to validate the presence and localization of CCR6^+^OLFM4^+^ stem cells in hypercoagulable versus normocoagulable CD patients.

### 2.11 Immunofluorescence staining

Immunofluorescence staining was performed on tissue sections fixed with 4% paraformaldehyde (15 min, RT), permeabilized with 0.1% Triton X-100 (10 min), and blocked with 5% BSA (1 h, RT). Samples were incubated with primary antibodies overnight at 4 °C, followed by species-matched secondary antibodies conjugated to FITC (1:400) and Cy3.5 (1:500) for 1 h (RT, dark). Nuclei were counterstained with DAPI (1 μg/mL, 5 min). After PBS washes, slides were mounted with [e.g., ProLong Gold] and imaged using a epifluorescence microscope with appropriate filters.

### 2.12 Statistical analysis

All statistical analyses were performed using R (v4.2.1) and GraphPad Prism (v10.1.2) with the following specific tests: (1) Differential gene expression analysis used limma with Benjamini-Hochberg correction; (2) Correlations employed Spearman’s rank test; (3) Group comparisons used Wilcoxon rank-sum (Mann-Whitney) for two groups, and Student’s t-test for parametric data; (4) Single-cell analyses applied MAST framework with FDR correction; (5) Immune infiltration used CIBERSORT’s built-in linear support vector regression with *P*<0.05 cutoff. All tests were two-tailed with α=0.05, and multiple testing corrections were applied where appropriate. *P* < 0.05 was considered statistically significant.

## 3 Results

### 3.1 Clinical evaluation and association between coagulation parameters, inflammatory markers, and disease activity in Crohn’s disease

**Figure 2:**
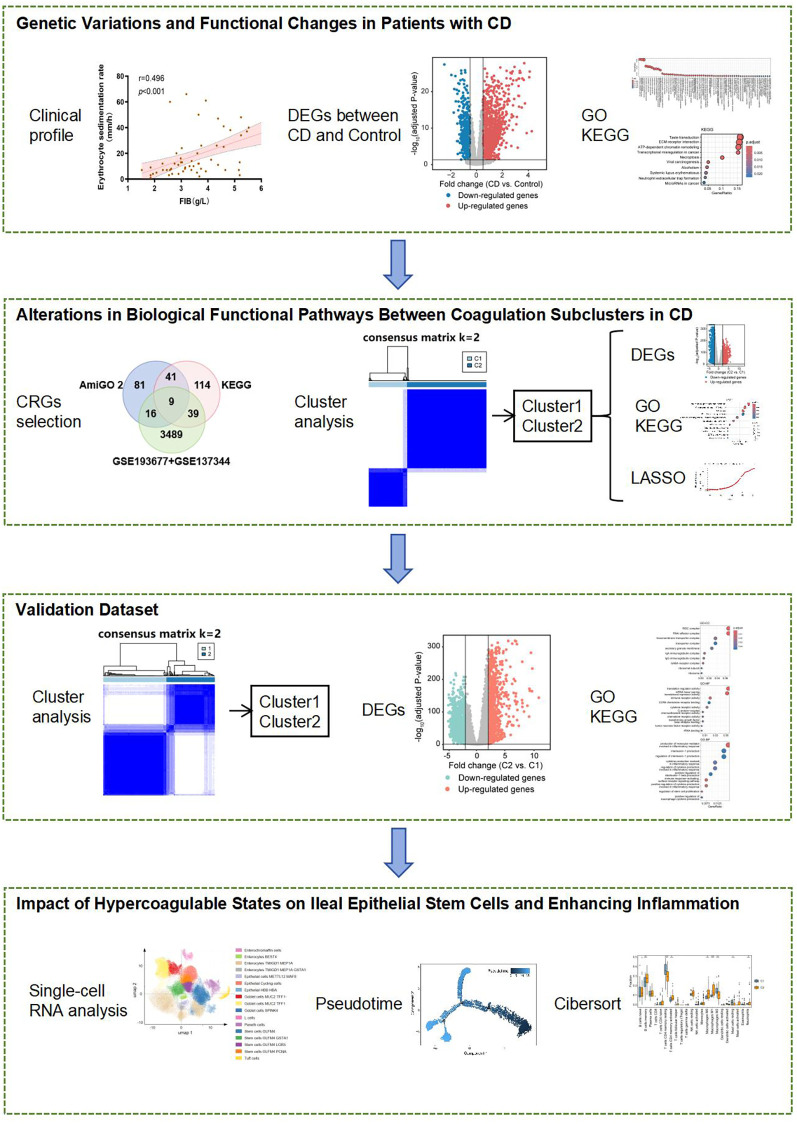
The flowchart for this study.

The flowchart of our study is illustrated in **Figure 2**.

To bridge clinical observations with molecular mechanisms, we employed a dual-strategy: (1) Clinical cohort validated associations between coagulation markers and disease severity; (2) GEO datasets powered differential expression and pathway analyses. This design ensured clinical relevance while maximizing genomic insights.

First, a total of 78 Crohn’s disease (CD) patients were included in this study, and their clinical information was collected. In addition to basic demographic data, detailed information such as inflammatory markers, CDAI scores, ultrasound reports, medication history, and coagulation-related parameters was recorded (**Supplementary Table 1**).

Initially, we assessed the inflammatory markers in CD patients and found that C-reactive protein (CRP) (mean ± s.d.: 53.7 ± 68.400, normal range: 0.00–6.00 mg/L), procalcitonin (ProCT) (mean ± s.d.: 0.6 ± 2.557, normal range: 0.000–0.050 ng/mL), and erythrocyte sedimentation rate (ESR) (mean ± s.d.: 19.6 ± 17.168, normal range: 0–15 mm/1h) were elevated. The elevation of these inflammatory markers indicates systemic inflammation and disease progression in CD patients.

Subsequently, we performed a correlation analysis between coagulation-related parameters and inflammatory markers. Platelet count was positively correlated with the Crohn’s Disease Activity Index (CDAI) (r = 0.281, p = 0.013) (**Figure 1A**) and ESR (r = 0.424, p <0.001) (**Figure 1B**). While the correlation between platelet count and CDAI was modest, its statistical significance suggests that coagulation parameters may serve as ancillary indicators of disease activity. In addition to platelet count, D-dimer levels and fibrinogen (FIB) levels were also positively correlated with ESR (r = 0.399, p = 0.002 and r = 0.496, p < 0.001, respectively) (**Figures 1C, D**).

Furthermore, patients were stratified into inactive, mild, and moderate groups based on CDAI scores. A comparison of platelet counts, D-dimer levels, and fibrinogen (FIB) levels among these groups revealed that platelet counts were significantly higher in the moderate group compared to the inactive group (**Figure 1E**). Additionally, both D-dimer levels and fibrinogen (FIB) levels increased progressively with disease severity (**Figures 1F, G**).

### 3.2 Genetic variations and functional changes in patients with Crohn’s disease

**Figure 3:**
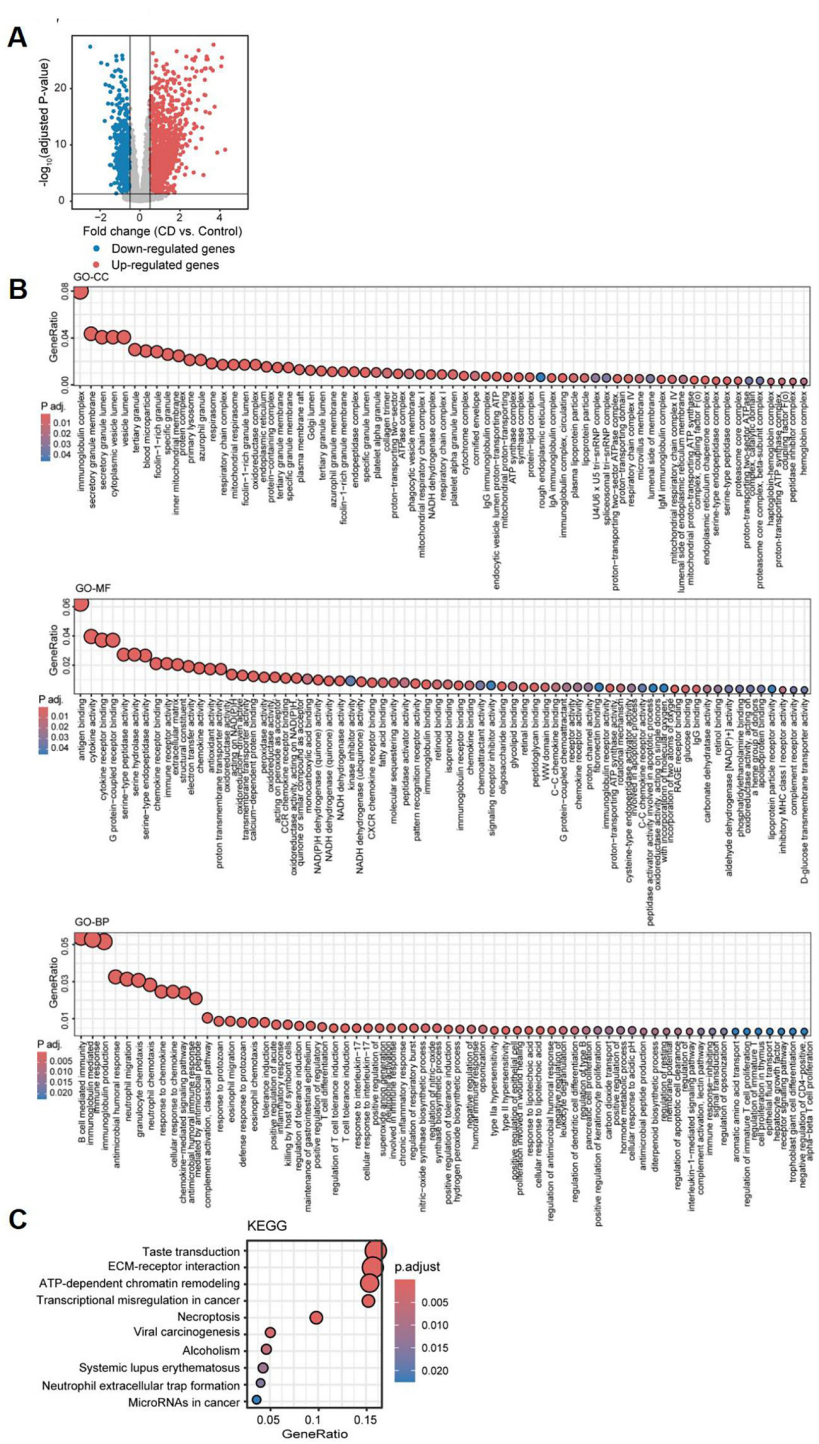
Differential gene expression analysis and functional pathway enrichment of ileal samples from Crohn’s disease patients compared to healthy controls. **(A)** Volcano plot depicting the log2 fold change and *P*-value for DEGs in CD patients compared to healthy controls. Up- and down-regulated genes are shown in red and blue, respectively. Genes with |logFC| ≤ 0.5, and adjusted *P*-value ≥ 0.05 are considered as insignificant genes, and are represented in grey. Function and pathway enrichment analysis results of DEGs across the three Gene Ontology categories: Cellular Component (GO-CC; top), Molecular Function (GO-MF; middle), and Biological Process (GO-BP; bottom) **(B)**, and the KEGG database **(C)**.

Based on clinical observations, we aimed to investigate the association between coagulation and the ileal type of CD, as well as the potential role of coagulation in the development and progression of this subtype. To achieve this, we analyzed a discovery dataset consisting of 234 ileal CD samples and 142 control samples, integrating data from GSE137344 and GSE193677. We identified a total of 3,553 differentially expressed genes (DEGs), including 2,247 upregulated and 1,306 downregulated genes, using a threshold of |logFC| > 0.5 and an adjusted *P* value < 0.05 (**Figure 3A**). To further investigate the potential biological roles of these DEGs, we performed Gene Ontology (GO) and Kyoto Encyclopedia of Genes and Genomes (KEGG) enrichment analyses utilizing the “cluster Profiler” package in R software. The GO enrichment analysis highlighted significant pathways, including immunoglobulin complex, production of molecular mediator of immune response, immunoglobulin mediated immune response, chemokine-mediated signaling pathway, regulation of inflammatory response, and blood coagulation. Additionally, in terms of molecular function, we identified significant activities related to chemokine activity, cytokine activity, CCR chemokine receptor binding, and immunoglobulin receptor binding (**Figure 3B**). Moreover, the KEGG analysis indicated that the DEGs were significantly enriched in pathways such as neutrophil extracellular trap formation, transcriptional misregulation in cancer, and microRNAs in cancer (**Figure 3C**). These findings collectively demonstrate the activation of immune infiltration and coagulation pathways in CD patients.

### 3.3 Variations in disease severity among CD patients based on coagulation scores

**Figure 4:**
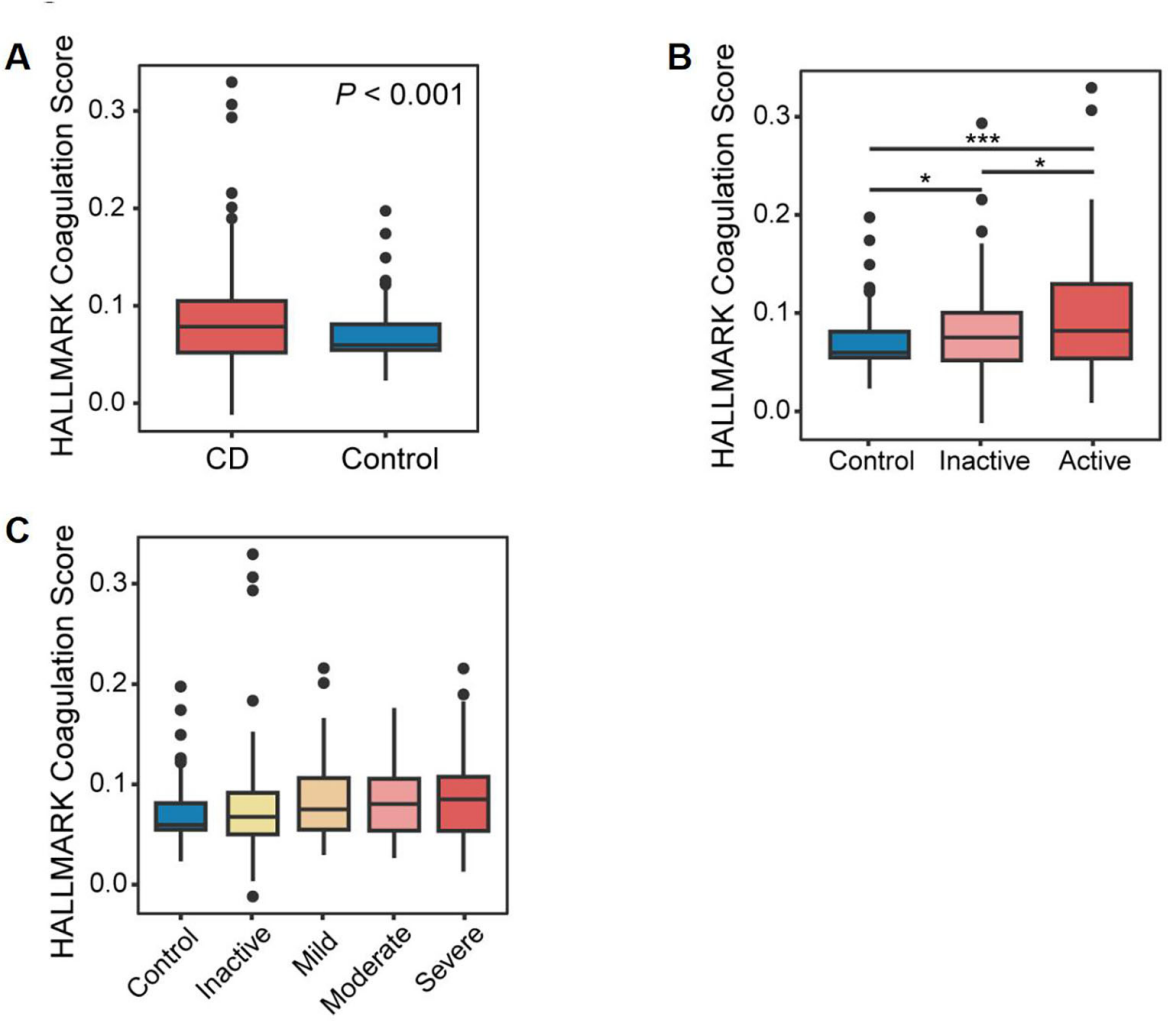
Identification of coagulation-related genes. **(A)** Box plots demonstrating a statistically significant difference in coagulation scores in CD patients compared to controls. **(B)** Box plots showing a statistically significant difference in coagulation scores among healthy controls, patients with inactive CD, and those with active CD. **(C)** Box plots illustrating a statistically significant difference in coagulation scores across disease activity levels, as defined by standardized clinical and endoscopic severity assessments.*P<0.05 and ***P<0.001.

We applied Gene Set Variation Analysis (GSVA) using the MSigDB “HALLMARK_COAGULATION” set, which provided a standardized metric of coagulation pathway activity. This score not only differed significantly between non-CD and CD tissues—including large cohorts with endoscopically defined inflamed and non-inflamed intestinal samples—but was also correlated with clinical indices and used to differentiate molecular subgroups (**Figure 4A**). Additionally, we examined the differences in coagulation scores across various “clinical and endoscopic definitions of disease activity,” including the clinician-based Harvey-Bradshaw index and endoscopic severity assessments. Notably, coagulation scores were consistently elevated in CD tissues and progressively increased in accordance with disease activity, further suggesting its utility as a quantitative severity marker (**Figures 4B, C**).

**Figure 5:**
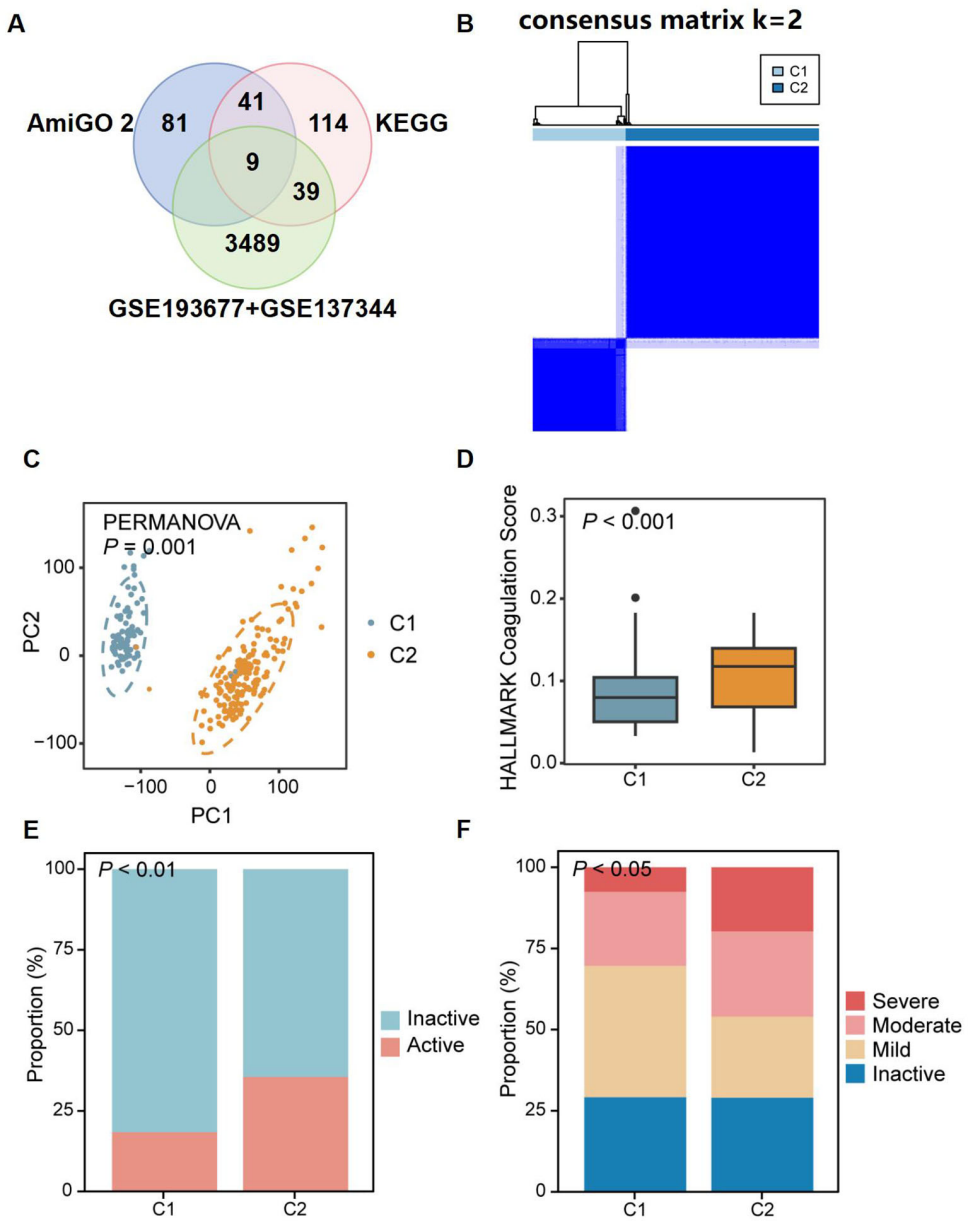
Identification of two distinct CD patient subclusters based on the expression of coagulation-related genes. **(A)** Venn diagram showing the overlap between coagulation-related genes from AmiGO 2 and KEGG databases and DEGs identified in discovery dataset. **(B)** 64 CRGs distinguished two subclusters of CD patients: Cluster 1 (C1) and Cluster 2 (C2). The two CD subclusters exhibited significant gene expression differences **(C)**, with C2 showing higher coagulation scores **(D)** and more severe disease **(E, F)**. P<0.05, P<0.01, and P<0.001.

Coagulation-related genes (CRGs) were sourced from the AmiGO 2 and KEGG databases and compared with the discovery datasets, yielding 64 overlapping CRGs (**Figure 5A**), which were subsequently used to stratify patients in order to capture coagulation-driven heterogeneity within CD. This step allowed us to test whether distinct coagulation-associated molecular patterns correspond to differences in clinical severity. Two distinct regulatory patterns were identified using an unsupervised clustering method, comprising 76 cases in coagulation-related Cluster 1 and 158 cases in Cluster 2 (**Figure 5B**). Principal Component Analysis (PCA) revealed a clear separation of patients into two groups, further confirming the existence of these distinct subtypes based on genetic profiling (**Figure 5C**). Additionally, we observed that Cluster 2 had higher “coagulation” scores (**Figure 5D**) and greater disease severity compared to Cluster 1 (**Figures 5E, F**). These findings suggest that coagulation may play a significant role in influencing disease progression and severity in patients with CD.

### 3.4 Alterations in biological functional pathways between coagulation subclusters

**Figure 6:**
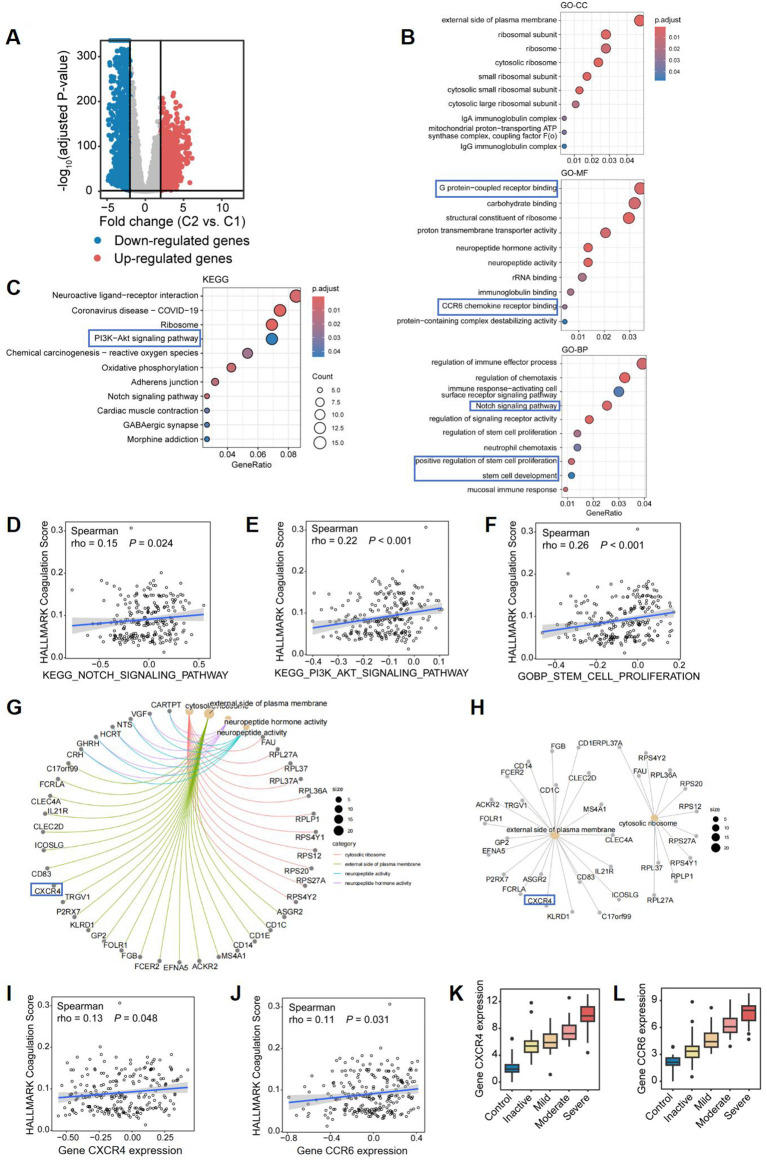
Differential gene expression and pathway enrichment analysis comparing two CD subclusters. **(A)** Volcano plot of differentially expressed genes (DEGs) between Cluster 2 (high coagulation) vs Cluster 1 (low coagulation) (|logFC|≥0.5, adj. p<0.05). Red: 1,061 upregulated; Blue: 2,755 downregulated. **(B, C)** Functional enrichment of DEGs: **(B)** Top 10 enriched GO terms (Cellular Component, Molecular Function, Biological Process); **(C)** Top 10 KEGG pathways. **(D–F)** Spearman correlations between coagulation scores and: **(D)** Notch signaling pathway activity; **(E)** PI3K-Akt signaling pathway; **(F)** stem cell proliferation signature. **(G–I)** Protein-protein interaction networks of shared genes across: **(G)** All enriched GO terms; **(H)**Cellular Component terms. The correlation of coagulation score and **(I)** *CXCR4* expression and **(J)** *CCR6* expression, respectively. Box plots of **(K)** *CXCR4* expression and **(L)** *CCR6* expression among groups.

To explore the potential factors influencing disease progression and severity in CD patients with elevated coagulation scores, we conducted a differential analysis. A total of 3,816 DEGs were identified between Cluster 1 and Cluster 2, including 1,061 upregulated genes and 2,755 downregulated genes in Cluster 2, with a threshold of |logFC| > 0.5 and an adjusted *P* value of < 0.05 (**Figure 6A**). The GO enrichment analysis highlighted significant pathways enriched in Cluster 2, such as IgA and IgG immunoglobulin complex, ribosome and external side of plasma membrane, G protein-coupled receptor binding, CCR6 chemokine receptor binding, and rRNA binding. Additionally, in terms of biological process, we identified significant activities related to Notch signaling pathway, mucosal immune response, regulation of immune effector processes, immune response-activating cell surface receptor signaling pathway, positive regulation of stem cell proliferation, and stem cell development (**Figure 6B**). Furthermore, the KEGG analysis indicated that the DEGs were significantly enriched in pathways such as PI3K-Akt signaling pathway, Notch signaling pathway, ribosome and oxidative phosphorylation (**Figure 6C**). These results demonstrate that in a subcluser of CD patients with higher coagulation scores, immune response and stem cell proliferation pathways were activated in ileal tissue. Similarly, we performed Gene Set Variation Analysis (GSVA) on the transcriptomic data to conduct functional enrichment analyses using the KEGG and GO databases (**Supplementary Figures S1A, B**). Based on the results of the functional enrichment, we also found that Notch signaling pathway(rho=0.15 p=0.024), PI3K-Akt signaling pathway (rho=0.22 p<0.001), and stem cell proliferation (rho=0.26 p<0.001) exhibited a significant positive correlation with coagulation scores in patients with CD (**Figures 6D–F**). Multiple studies demonstrate that the Notch signaling pathway regulates inflammatory cell differentiation (16) and modulates PI3K-Akt signaling (17). Given the established role of PI3K-Akt, downstream of Kit (18), in cell proliferation, survival, and metabolism (19), we hypothesize that increased coagulation scores in CD patients result in Notch-mediated activation of PI3K-Akt, thereby promoting intestinal epithelial stem cell proliferation and differentiation. Overall, the functional pathway enrichment in Cluster 2 was primarily centered on cytoplasmic ribosomes and associated genes involved in cytokine signaling, including *CXCR4*, a G protein-coupled receptor of the same class as *CCR6* (**Figure 6G**). Similarly, we found that gene *CXCR4* and *CCR6* exhibited a significant positive correlation with coagulation scores in patients with CD (**Figures 6I, J**), and both of them shows a trend of high expression as the severity of CD increases (**Figures 6K, L**). Studies indicate that chemokine-G protein-coupled receptor interactions can activate the PI3K-Akt signaling pathway (20, 21). Therefore, we hypothesize that in CD patients with hypercoagulability, increased expression of CXCR4 and CCR6 chemokine receptors in ileal tissue may, through interaction, synergistically activate and modulate PI3K-Akt signaling with the Notch signaling pathway, promoting intestinal epithelial stem cell proliferation and differentiation, and thus influencing disease severity and progression.

Furthermore, all genes were included in the LASSO regression analysis, and 12 genes were identified as potential biomarkers for distinguishing CD patients with varying coagulation states (**Supplementary Figures S2A, B**). Among these 12 critical genes (**Supplementary Table S1**), AKT1, CCR6, and CXCR4 further corroborated the plausibility and significance of our previously proposed hypothesis. Additionally, the ROC curve for the diagnostic model based on these 12 key genes yielded an AUC value exceeding 0.6 (AUC = 0.887, **Supplementary Figure S2C**), indicating the valuable diagnostic performance of the model. This model effectively distinguishes CD patients with different coagulation states and further validates the importance of the expression of CXCR4 and CCR6 chemokine receptors, along with the activation of the PI3K-Akt signaling pathway, in influencing the progression of CD.

### 3.5 Coagulation shows correlation with CD progression through the PI3K-Akt signaling pathway in the validation dataset

**Figure 7:**
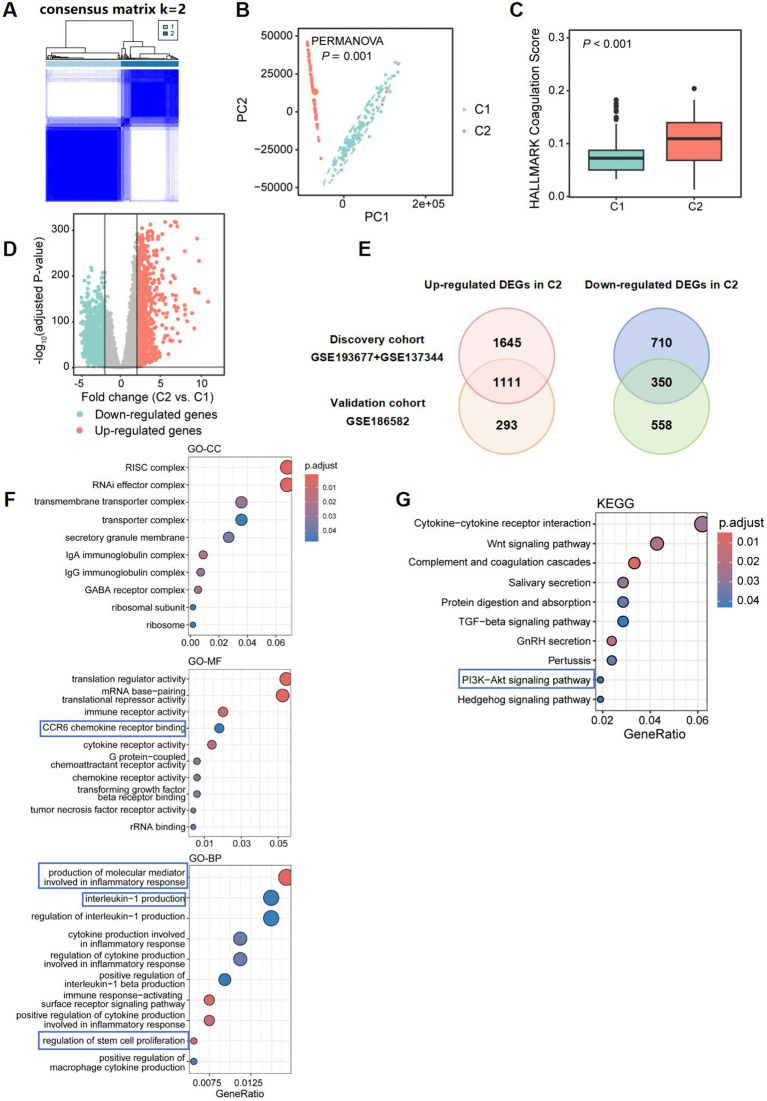
Differential gene expression and pathway enrichment analysis comparing two CD subclusters in validation dataset. **(A)** 64 CRGs distinguished two subclusters of CD patients in validation dataset: Cluster 1 (C1) and Cluster 2 (C2). The two CD subclusters exhibited significant gene expression differences **(B)**, with C2 showing higher coagulation scores **(C)**. **(D)** Volcano plot depicting the log2 fold change and *P*-value for DEGs in Cluster 2 compared to Cluster 1. Up- and down-regulated genes are shown in light red and light blue, respectively. Genes with |logFC| ≤ 0.5, and adjusted *P*-value ≥ 0.05 are considered as insignificant genes, and are represented in grey. **(E)** Venn diagram showing overlap of differentially expressed genes (DEGs) in discovery and validation datasets. Top 10 enriched GO terms [Cellular Component (GO-CC; top), Molecular Function (GO-MF; middle), Biological Process (GO-BP; bottom)] and KEGG pathways associated with immune and inflammatory responses, identified from function and pathway enrichment analysis **(F, G)**.

To validate the robustness of our findings, we utilized the dataset GSE186582 as an additional validation set. Similarly, employing 64 DEGs, we classified the CD patients in the validation cohort into two clusters: Cluster 1, comprising 111 cases related to coagulation, and Cluster 2, consisting of 85 cases (**Figure 7A**). These two groups also exhibited significant differences at the gene level (**Figure 7B**), with Cluster 2 exhibiting higher “coagulation” scores (**Figure 7C**). After filtering low-expressing genes, a total of 20,049 genes were retained. A total of 2,312 DEGs were identified between Cluster 1 and Cluster 2, including 1,061 upregulated genes and 2,755 downregulated genes in Cluster 2, with a threshold of |logFC| > 0.5 and an adjusted *P* value of < 0.05 (**Figure 7D**). In the validation dataset, we found 1,111 upregulated DEGs that overlapped with those from the discovery dataset, resulting in a consistency rate of 79.13%, thereby confirming the robustness of our results (**Figure 7E**).

The pathway enrichment analysis of DEGs in GO revealed that Cluster 2 showed consistent enrichment for functions related to immunoglobulin complexes and ribosomes, IgA and IgG immunoglobulin complex, as well as cytokine receptor activity, G protein-coupled chemoattractant receptor activity, and CCR6 chemokine receptor binding with the discovery dataset. It also demonstrated enrichment in pathways regulating stem cell proliferation when compared to the discovery dataset. Furthermore, pathways involved in the production of molecular mediators that participate in inflammatory responses and the activation of immune signaling pathways regulating interleukin-1 production were identified (**Figure 7F**) KEGG analysis revealed additional enrichment of DEGs in the PI3K-Akt signaling pathway, underscoring the significant role this pathway plays in the progression of CD in hypercoagulable states (**Figure 7G**).

### 3.6 Impact of hypercoagulable states on ileal epithelial stem cells in CD patients

The intestinal barrier consists of a monolayer of specialized intestinal epithelial cells, which play a critical role in maintaining mucosal homeostasis. In our study, we observed that elevated coagulation scores in CD patients were significantly associated with increased proliferation and development of stem cells. Furthermore, excessive intestinal epithelial stem cell proliferation may exacerbate colitis severity (22). These findings suggest that coagulation may impact the progression of CD by modulating the dynamics of intestinal epithelial stem cells.

**Figure 8:**
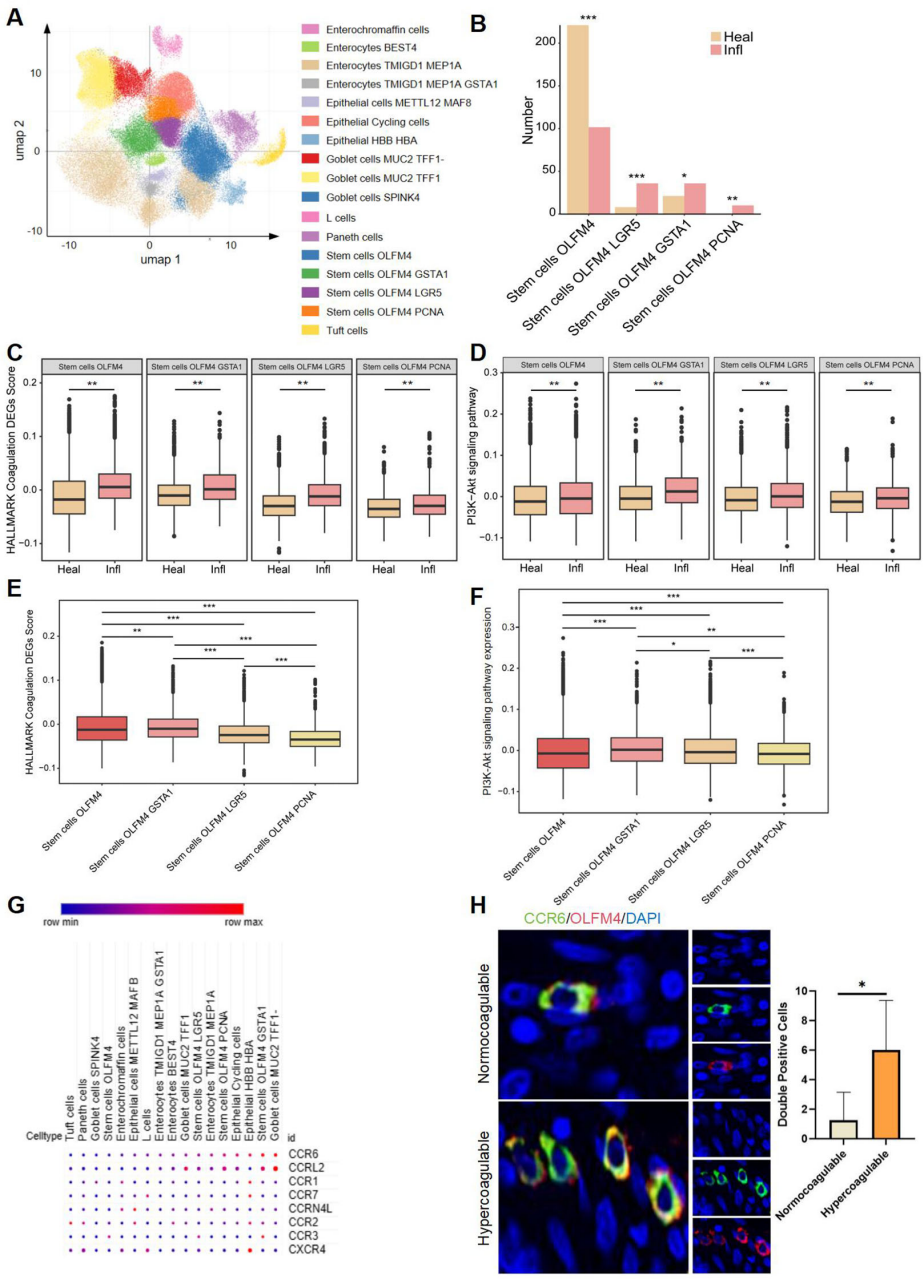
CCR6^+^OLFM4^+^ stem cell expansion in hypercoagulable CD. **(A)** UMAP visualization of epithelial cells in ileal tissue, colored by detailed cell types within compartments. **(B)** The bar plot presents the number of the four stem cell subtypes between the two groups. Box plots showing higher coagulation scores **(C)** and PI3K-Akt pathway expression **(D)** in stem cell of inflamed ileal tissue from CD patients. Box plots showing different coagulation scores **(E)** and PI3K-Akt pathway expression **(F)** in different stem cell subtypes of inflamed ileal tissue from CD patients. **(G)** Heatmap showing the differential expression of G protein-coupled chemokine receptors in various cell subtypes. Red denotes up-regulation; blue denotes down-regulation. **(H)** Differences in CCR6^+^OLFM4^+^ stem cells stained for CCR6 (green) and OLFM4 (red) and DAPI (blue) among patients with CD in normocoagulable and hypercoagulable.*P<0.05, **P<0.01, and ***P<0.001.

To further investigate this, we analyzed single-cell transcriptomic data from 154,136 epithelial cells derived from the ileal region in publicly available databases (SCP1884) (15). Through clustering and annotation, we identified 17 distinct cell types, including four stem cell subtypes: stem cells OLFM4, stem cells OLFM4 GSTA1, stem cells OLFM4 LGR5, and stem cells OLFM4 PCNA (**Figure 8A**). Among these, the other three stem cell subtypes, excluding stem cells OLFM4, were expressed at significantly higher levels in the inflammatory samples from patients with CD (**Figure 8B**). The gene *OLFM4* serves as a reliable marker for stem cells in the human gut and can identify a subset of colorectal cancer cells. *GSTA1* has been found to be overexpressed in colon cancer, while *LGR5* is expressed in various human cancers and colorectal cancer stem cells. Additionally, *PCNA*, which is present only in normally proliferating and tumor cells, is an excellent indicator of cellular proliferation. All four identified subtypes are associated with colorectal cancer, and their gene expressions are linked to cell proliferation and cancer progression, consistent with established links between stem cell hyperproliferation and colorectal carcinogenesis (23).

Through GSVA analysis, we found that the four stem cell subtypes in the diseased intestines of CD patients exhibited elevated coagulation scores and increased expression of the PI3K-Akt signaling pathway. These findings suggest that a hypercoagulable state may affect the function and differentiation of stem cells via the PI3K-Akt signaling pathway (**Figures 8C, D**), thereby influencing the progression of ileal CD and potentially contributing to intestinal cancer development. Among the four stem cell subtypes, stem cells OLFM4 GSTA1 exhibited the highest coagulation score and significantly increased expression of the PI3K-Akt signaling pathway (**Figures 8E, F**). Additionally, the chemokine CCR6 was prominently up-regulated in stem cells OLFM4 GSTA1 (**Figure 8G**). This finding is consistent with the literature indicating that the binding of *CCR6* to its ligand can activate the PI3K/Akt pathway, thereby triggering downstream protein kinases and initiating a cascade reaction that promotes the expression of corresponding chemokines, leading to cellular migration and metastasis (24, 25). Immunofluorescence staining of ileal tissues provided direct histological validation, demonstrating significantly increased CCR6^+^OLFM4^+^ double-positive cells in the epithelial layer of hypercoagulable CD patients compared to normocoagulable controls (*P*<0.05, **Figure 8H**). These results suggest CCR6 as a potential mediator linking hypercoagulability to PI3K-Akt pathway activity in ileal epithelial stem cells, thereby impacting the progression of CD.

**Figure 9:**
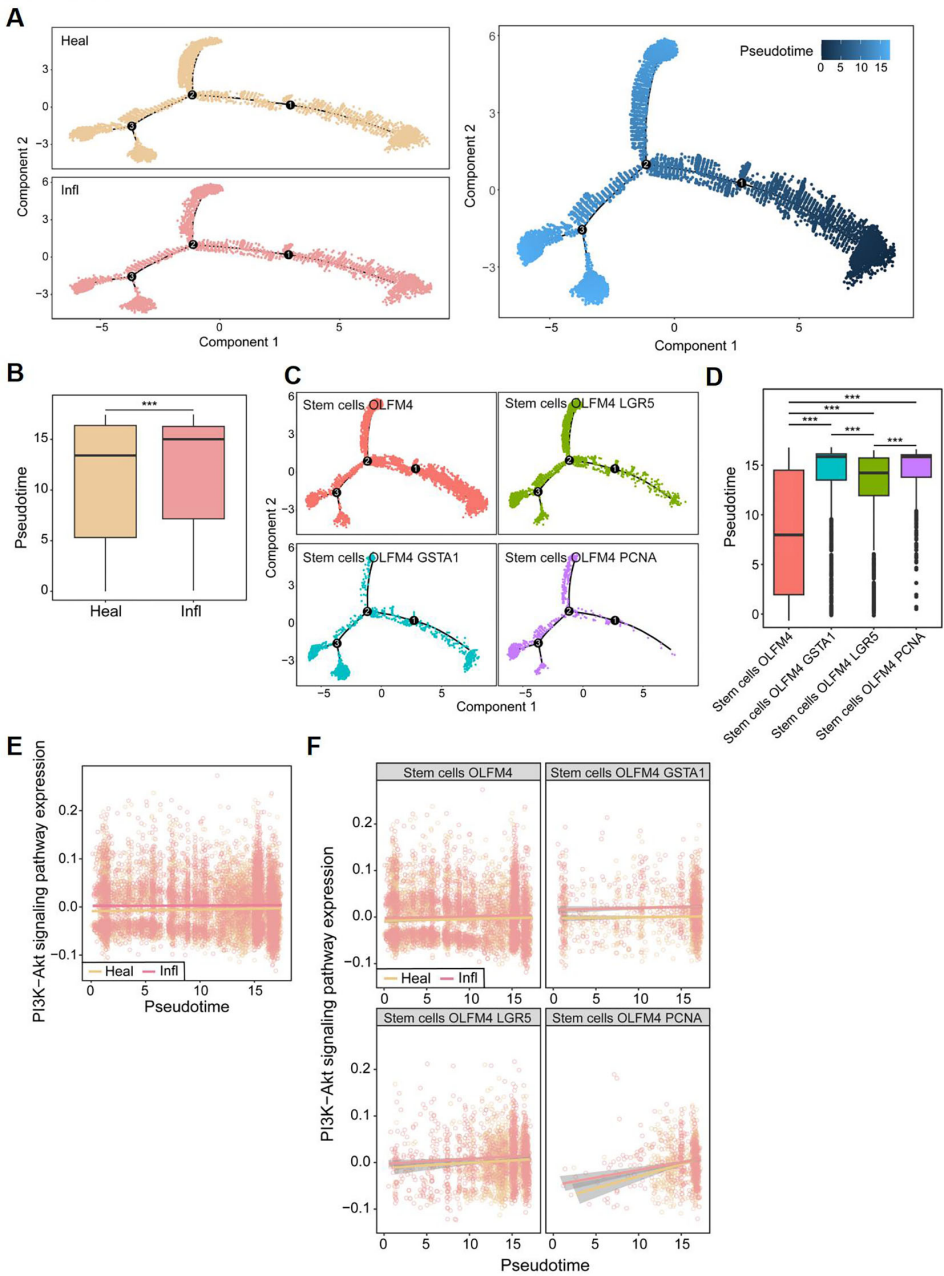
Pseudo-temporal analysis in single cells. **(A)** The single-cell trajectory map for two subclusters. **(B)** Box plots showing differences in Pseudo-time between CD patients (Infl) and healthy control (Heal). **(C)** The single-cell trajectory map for four stem cell subtypes. **(D)** Box plots showing differences in Pseudo-time among stem cell subtypes. **(E)** Pseudo-temporal trajectories for PI3K-Akt signaling pathway between CD patients and healthy control. **(F)** Pseudo-temporal trajectories for PI3K-Akt signaling pathway between CD patients and healthy control in four stem cell subtypes, respectively. *P<0.05, **P<0.01, and ***P<0.001.

Moreover, the single-cell trajectory analysis demonstrated distinct levels of stem cell differentiation between CD patients and healthy controls (**Figure 9A**), with CD patients exhibiting significantly higher degrees of cell differentiation (**Figure 9B**). The trajectory analysis of the four total stem cell subtypes indicated that stem cells OLFM4 is the earliest differentiating cell, gradually proliferating and differentiating into subtypes excluding stem cells OLFM4 GSTA1, stem cells OLFM4 LGR5, and stem cells OLFM4 PCNA (**Figures 9C, D**). Combined with our earlier findings, which showed that, compared to healthy controls, stem cells OLFM4 GSTA1, stem cells OLFM4 LGR5, and stem cells OLFM4 PCNA were enriched in the inflammatory intestinal epithelium of patients with CD (**Figure 8B**), this result further highlights the critical role of stem cell proliferation and differentiation in CD patients. The pseudotemporal analysis revealed a consistent upward trajectory in PI3K-Akt pathway activity during stem cell differentiation (**Figure 9E**). While absolute expression differences between healthy and inflamed samples were modest, the progressive activation pattern across differentiation stages suggests PI3K-Akt may play a role in coordinating stem cell fate decisions (**Figure 9F**). This observation aligns with established mechanisms of PI3K-Akt in progenitor cell regulation (19), and further corroborates that the activation of the PI3K-Akt signaling pathway may lead to excessive differentiation of stem cells, thereby exacerbating the progression of CD.

### 3.7 Impact of hypercoagulable states on enhancing inflammation in CD patients

**Figure 10:**
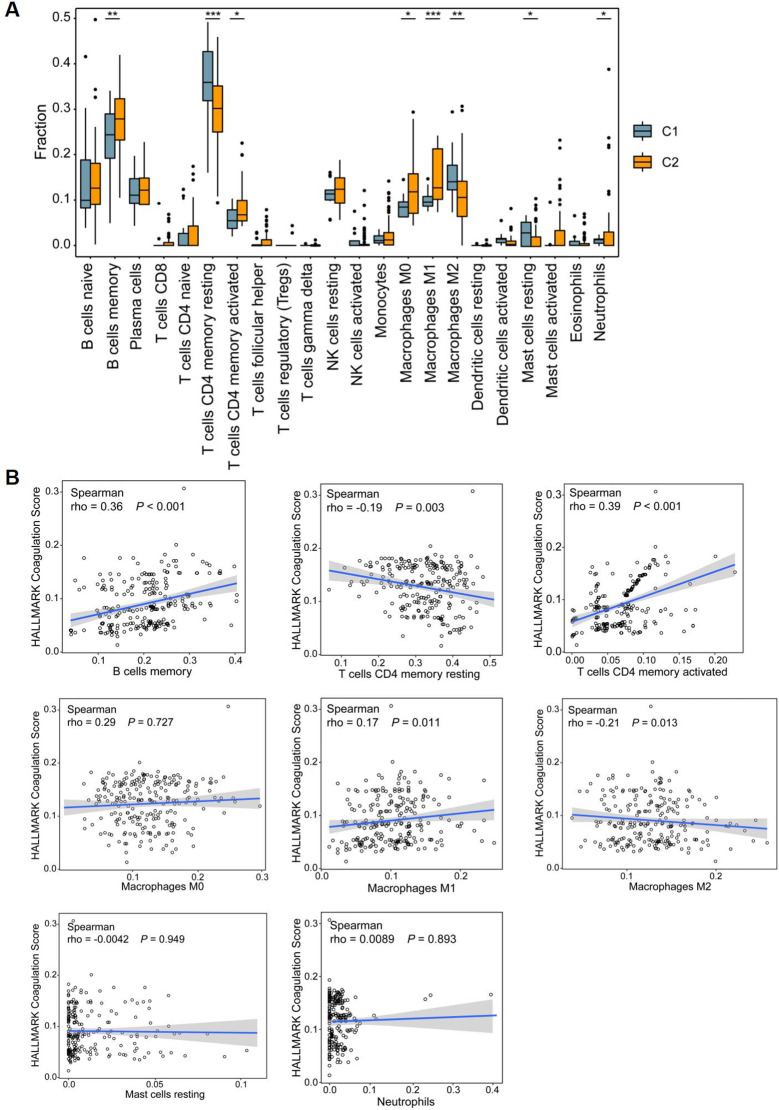
Immune microenvironment among CD patients. **(A)** Box plots showing differences in immune infiltration between C1 and C2 subclusters in the discovery dataset. **(B)** The correlation of coagulation score and differential immune checkpoints. *P<0.05, **P<0.01, and ***P<0.001.

In CD patients, the intestinal mucosa exhibits characteristic fissuring ulcers. During the repair of these lesions, intestinal stem cells play a crucial role through their active proliferation and differentiation to restore epithelial integrity. In parallel, marked inflammatory cell infiltration is consistently observed in the mucosa of CD patients, representing another key pathological feature. Building on our earlier findings that hypercoagulable states were associated with enrichment of inflammatory signaling pathways, such as IL-1, we next sought to investigate immune cell infiltration in greater detail. To directly connect these observations with our subgroup analyses, we examined whether differences in immune infiltration profiles aligned with the coagulation-related clusters identified earlier. Since *CCR6* is also associated with a variety of inflammatory diseases (26), including Colitis-associated carcinoma (27), and we observed significant upregulation of immune responses and immune cell activation in CD patients exhibiting a hypercoagulable state (**Figure 10A**), we investigated immune cell infiltration investigation to further elucidate the immunomodulatory effects of coagulation in CD. Box plots indicated that the C2 group displayed higher proportions of memory B cells, memory-activated CD4 T cells, M0 and M1 macrophages, and neutrophils. In contrast, the proportions of memory resting CD4 T cells, M2 macrophages, and resting mast cells were decreased. Meanwhile, spearman correlation analysis indicated a significant association between coagulation scores and these eight differential immune cells (**Figure 10B**). Specifically, memory B cells (rho=0.36, p<0.001), memory-activated CD4 T cells (rho=0.39, p<0.001), and M1 macrophages(rho=0.17, p=0.011) showed positive correlations with the coagulation score, while memory-resting CD4 T cells (rho=-0.19, p=0.003) and M2 macrophages (rho=-0.21, p=0.013) exhibited negative correlations. The infiltration of different types of immune cells in patients with celiac disease, along with their distinct associations with the coagulation score, may represent another potential therapeutic target for CD.

## 4 Discussion

Our study identifies CCR6^+^OLFM4^+^ intestinal stem cells as a previously unrecognized cellular interface between coagulation and inflammation in Crohn’s disease. Three lines of evidence support this conclusion: (1) the selective expansion of this stem cell subpopulation in hypercoagulable ileal tissues, (2) their co-localization with microthrombotic lesions (**Figure 8H**), and (3) their unique transcriptional signature featuring concurrent CCR6 overexpression and PI3K-Akt pathway activation. These findings position CCR6^+^OLFM4^+^ cells as potential effectors linking coagulation abnormalities to disease progression.

The mechanistic plausibility of this association stems from the CCR6-CCL20 axis’ known role in epithelial pathophysiology. Our single-cell data demonstrate that CCR6^+^OLFM4^+^ cells exhibit PI3K-Akt activation signatures similar to those observed in CCR6-mediated chemotaxis (27–33). This is particularly noteworthy given that PI3K-Akt signaling promotes stem cell survival and proliferation (19, 20, 34) – processes we found enriched in hypercoagulable patients. We propose a model whereby coagulation factors (e.g., thrombin or fibrinogen) create a permissive microenvironment for CCR6^+^ stem cell expansion, which in turn amplifies local inflammation through PI3K-Akt-driven cytokine production.

These observations have immediate translational implications. First, they suggest that existing anticoagulants may exert previously unrecognized benefits in CD by modulating the intestinal stem cell niche (35–37). Second, they identify CCR6 as a potential therapeutic target, with several CCR6 inhibitors currently in clinical trials for other inflammatory conditions (26, 27, 38–41). Our data specifically support testing these agents in CD patients with elevated coagulation markers. Future studies should prioritize: (1) organoid models to test if CCR6 knockout abrogates coagulation-induced PI3K-Akt activation, and (2) clinical trials stratifying patients by coagulation status when evaluating CCR6-targeted therapies.

Several limitations warrant consideration. While we validated our findings across multiple cohorts, the observational nature of our data cannot establish causality. The dual use of broad CRG screening (300 genes) and focused HALLMARK scoring represents a methodological strength but requires validation in prospective studies. Additionally, our single-cell analysis, while comprehensive, could be augmented by spatial transcriptomics to precisely map CCR6^+^OLFM4^+^ cells relative to thrombotic foci. Furthermore, while the pseudotemporal analysis revealed a consistent upward trajectory in PI3K-Akt pathway activity during stem cell differentiation, functional validation is needed to determine threshold effects for phenotypic changes. Future work should employ phospho-specific staining and live-cell imaging to precisely quantify Akt activation kinetics during differentiation.

In conclusion, this work advances our understanding of CD pathogenesis by revealing CCR6^+^OLFM4^+^ intestinal stem cells as cellular transducers of coagulation signals into pro-inflammatory responses. The CCR6-PI3K-Akt axis emerges as a unifying mechanism that could explain both the thrombotic complications and therapeutic responses to anticoagulation observed in CD patients. Further investigation of this axis may yield novel biomarkers for disease stratification and targeted therapies for this challenging condition.

## Data Availability

The datasets presented in this study can be found in online repositories. The names of the repository/repositories and accession number(s) can be found in the article/**Supplementary Material**.
