# Supplementary material for: Impact of hypercoagulable state on Crohn’s disease severity and progression: transcriptomic and single-cell analyses of the ileum
Source: Front Immunol. 2025 Sep 25;16:1611114. doi: 10.3389/fimmu.2025.1611114 (PMC12507747; doi:10.3389/fimmu.2025.1611114)
Supplement: Supplementary file 1 [file DataSheet1.pdf]

SUPPLEMENTAL MATERIAL TO

Impact of Hypercoagulable State on Crohn’s Disease Severity and Progression: Transcriptomic and Single-Cell Analyses of the Ileum

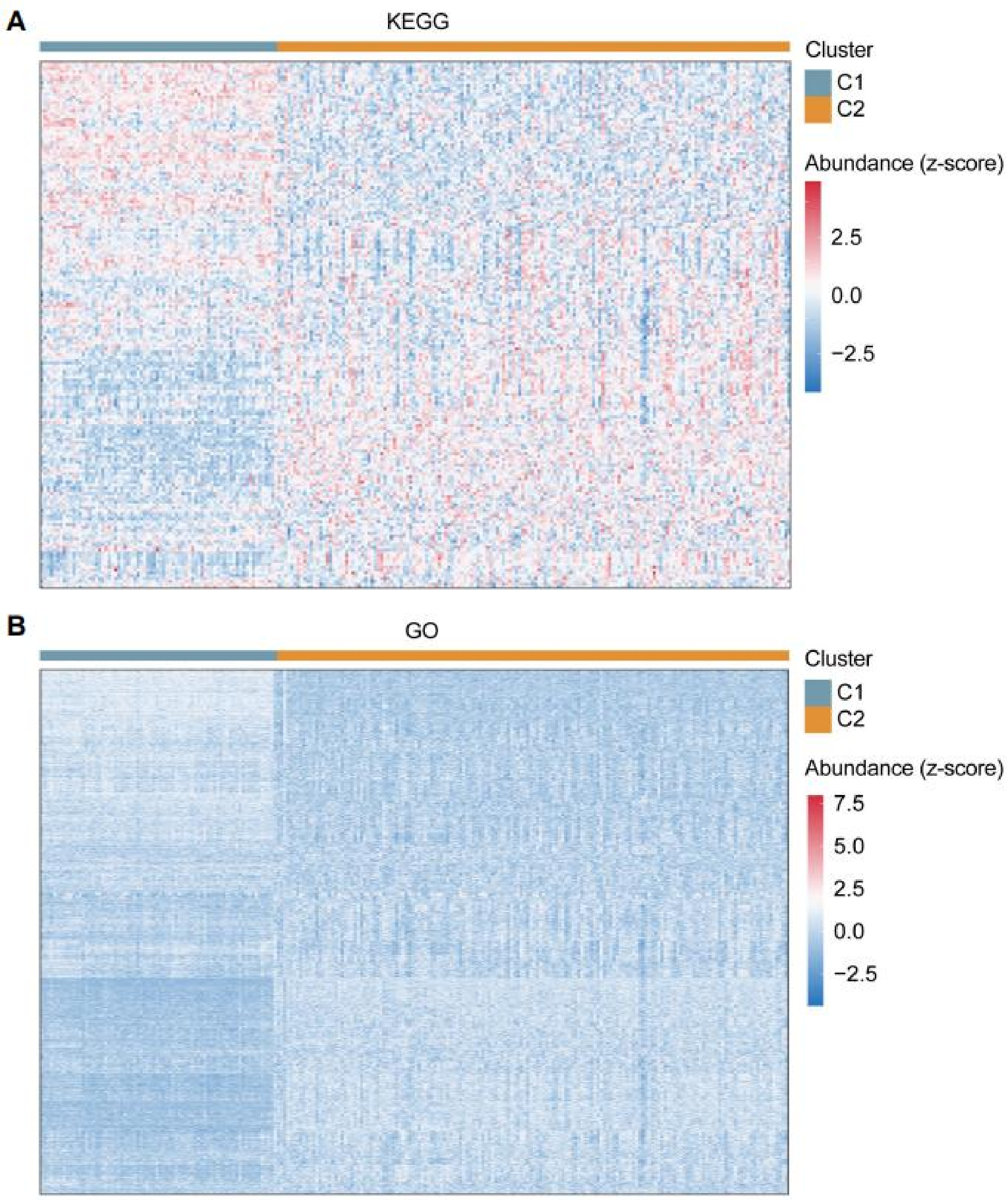

Figure S1. The heatmap of GSVA functional enrichment results is presented, related to Figure 6. GSVA results for functional enrichment based on the KEGG (A) and GO databases (B), respectively.

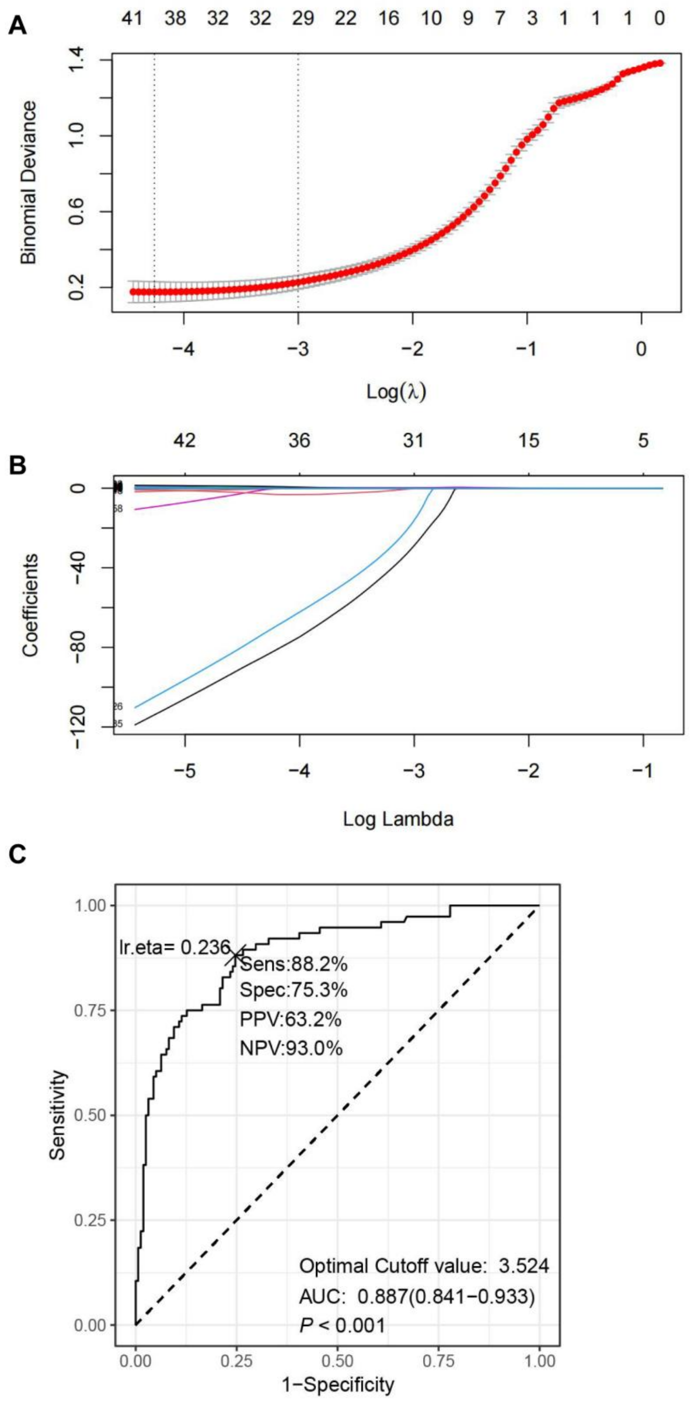

Figure S2. Construction of a model for distinguishing CD patients with higher coagulation states using machine learning approach. (A) Ten time cross-validation for biomarker selection in the LASSO model. (B) LASSO coefficient profiles. (C) ROC plot depicting the performance of the final model based on 40 biomarkers in the cohort.

Table S1. 12 biomarkers selected by the LASSO model.

Symbol	Description
AKT1	AKT serine/threonine kinase 1
CCR6	C-C motif chemokine receptor 6
GABRG1	gamma-aminobutyric acid type A receptor subunit gamma1
KRT6A	keratin 6A
CXCR4	C-X-C motif chemokine receptor 4
BSN	bassoon presynaptic cytomatrix protein
CLASRP	CLK4 associating serine/arginine rich protein
EXOC3	exocyst complex component 3
IGHVIII-44	immunoglobulin heavy variable (III)-44 (pseudogene)
IGHV3-75	immunoglobulin heavy variable 3-75 (pseudogene)
TRBV5-7	T cell receptor beta variable 5-7 (non-functional)
TRBJ2-1	T cell receptor beta joining 2-1
